# The Center for Health in Cognitive Aging (CHECA): A RCMAR Program Joining Population Science and Health Promotion

**DOI:** 10.1093/geroni/igaf122.578

**Published:** 2025-12-31

**Authors:** Uchechi Mitchell, Julia Bauer, Debaleena Chattopadhyay, Jennifer Kwok, Zhuoer Lin

**Affiliations:** University of Illinois Chicago, Chicago, Illinois, United States; University of Illinois Chicago, Chicago, Illinois, United States; University of Illinois Chicago, Chicago, Illinois, United States; University of Illinois Chicago, Chicago, Illinois, United States; University of Illinois Chicago, Chicago, Illinois, United States

## Abstract

The Center for Health in Cognitive Aging (CHECA) is a newly funded Resource Center for Minority Aging Research (RCMAR) at the University of Illinois Chicago. CHECA aims to build behavioral and social research infrastructure, diversify the AD/ADRD research workforce through mentorship and training of early-career Scientists, and support pilot research. The center thematically focuses on research that (1) advances our understanding of environmental, sociocultural, and biobehavioral contributors of AD/ADRD inequities; (2) develops or tests health-promoting interventions or resources; and (3) cross-fertilizes population science and health promotion to address inequities in adults with or at risk for AD/ADRD. This symposium highlights the exceptional research of four CHECA Scientists: Dr. Bauer’s research identified sex differences in the effects of persistent organic pollutants on biomarkers of neurodegeneration among middle-aged and older adults in the Hispanic Community Health Study. Dr. Chattopadhyay conducted mobile technology learning workshops with older adults with cognitive difficulties and identified challenges with digital security and usability that limited engagement with digital technologies. Dr. Kwok’s research used public use Census data and found racial/ethnic differences in living arrangements among older adults with cognitive difficulties that can influence access to informal caregiving. Lastly, using data from NHATS, Dr. Lin found that among older adults living with dementia, social isolation was positively associated with unmet care needs. Collectively, these projects advance AD/ADRD research across multiple levels of effect—biological, behavioral, familial, and social—and epitomize the interdisciplinary approaches needed to improve cognitive outcomes in diverse populations and address AD/ADRD inequities.

